# Hypogonadism in hemodialysis patients: a first snapshot of prevalence and predictive factors in Tunisian patients

**DOI:** 10.11604/pamj.2023.46.63.39794

**Published:** 2023-10-19

**Authors:** Nidhal Ati, Zohra El Ati, Ichrak Bannour, Amira Sallem, Amira Sghaier, Haifa Bouchahda, Baha Zantour, Hassen Bouzidi, Mohamed Yassine Binous

**Affiliations:** 1 Department of Urology, Tahar Sfar Hospital, Mahdia, Faculty of Medicine, Monastir University, Monastir, Tunisia,; 2 Department of Nephrology and Dialysis, Tahar Sfar Hospital, Mahdia, Research Unit, Applied Mental Health “UR12SP43”, Faculty of Medicine, Monastir University, Monastir, Tunisia,; 3 Immunology Laboratory, Fattouma Bourguiba University Hospital, University of Monastir, Monastir, Tunisia,; 4 Laboratory of Molecular Immuno-Oncology, Faculty of Medicine, Monastir University, Monastir, Tunisia,; 5 Laboratory of Histology Embryology and Cytogenetics (LR 40 ES 18), Faculty of Medicine, University of Monastir, Monastir, Tunisia,; 6 Laboratory of Cytogenetics and Reproductive Biology, Maternity and Neonatology Center, Fattouma Bourguiba University Teaching Hospital, Monastir, Tunisia,; 7 Emergency Department Tahar Sfar Hospital, Mahdia, Faculty of Medicine, Monastir University, Monastir, Tunisia,; 8 Department of Gynecology, Tahar Sfar Hospital, Mahdia, Faculty of Medicine, Monastir University, Monastir, Tunisia,; 9 Department of Endocrinology, Tahar Sfar Hospital, Mahdia, Faculty of Medicine, Monastir University, Monastir, Tunisia,; 10 Department of Biochemistry, Tahar Sfar Hospital, Mahdia, Tunisia

**Keywords:** Erectile dysfunction, hemodialysis, hypogonadism

## Abstract

**Introduction:**

patients with chronic kidney disease commonly exhibit testosterone deficiency. We aimed through the current study to assess the prevalence and the risk factors of hypogonadism in male patients on hemodialysis and to establish their relationship with erectile dysfunction.

**Methods:**

we conducted a cross-sectional study based on data collected from hemodialysis male patients. Sociodemographic and clinical data as well as hormone levels were collected from January 2017 to December 2017. Sex hormones were measured in all subjects. The International Index of Erectile Function was used to evaluate erectile dysfunction. Data were expressed as mean ± standard deviation, and frequencies (number), and proportions (%).

**Results:**

one hundred and ten: 55 male hemodialysis patients were recruited. The level of follicule-stimulating hormone, luteinizing hormone and prolactin were high and the level of testosterone was low in the hemodialysis group. Hypogonadism was significantly linked to advanced age, anemia, and absence of treatment by erythropoietin. The incidence of erectile dysfunction was high and the erectile function score was low. Testosterone significantly dropped in patients with erectile dysfunction.

**Conclusion:**

hypogonadism was so prevalent in the hemodialysis men and it was associated with erectile dysfunction. Future studies are needed to determine the effect of testosterone therapy on erectile dysfunction.

## Introduction

Sexual dysfunction in patients with chronic renal failure (CRF) is multifactorial. Disorders of the endocrine testicular function are factors that should be studied. Dialysis treatment has not been known to restore hormonal changes [1]. Hypothalamic-pitutary-gonadal axis disorders, secondary to alterations in feedback mechanisms and hormone production become clearer in patients receiving hemodialysis (HD) [2,3]. Previous studies have found high levels of prolactin [4,5] as well as luteinizing hormone (LH) and follicule-stimulating hormone (FSH) [5-7] and low levels of testosterone [8,9]. The testosterone deficiency is the most frequent gonadal disturbance in men with end-stage renal disease (ESRD), essentially due to reduced prolactin clearance [10] and uremic inhibition of LH [11]. It has a common systemic effect and has been associated with erectile dysfunction (ED) in dialysis patients.

The etiology is multifactorial, and the majority of patients have various comorbidities that can lead to hypogonadism [12]. Patients with ED are unable to complete and/or preserve a sufficient erection for appropriate sexual intercourse [13,14]. This could have a deleterious impact on the reproductive health of patients with chronic renal failure. In this study we will focus on several aspects of chronic renal failure-associated hypogonadism that remain unresolved, addressing the following questions: 1) What is the prevalence of hypogonadism in male patients undergoing HD? 2) What are the clinical and sociodemographic characteristics associated with HD?

The aims of the present study were to assess the prevalence of hypogonadism in male patients undergoing HD, study associations with risk factors, and determine their relationship with ED in HD.

## Methods

**Study design:** this was a cross-sectional study.

**Study size:** the sample size was calculated according to the BiostaTGV site [15]. It was based on a frequency of hypogonadism of 6% in the general population and an odds ratio of 6.19% in men with chronic renal failure [16]. Setting the power at 80% and the one-sided significance level at 0.05, 53 individuals were required in each group.

**Participants:** out of a cohort of 190 hemodialised patients in the period of January 2017 to December 2017, we included in the current study all male HD patients for more than six months and aged between 18 and 60 years. All patients required regular HD sessions for 4 hours, three times a week. Standard heparin was administered before each HD session. Blood flow was usually 300 mL/min with a dialysate flow at a rate of 500 mL/min. Patients were dialyzed with high-flux polysulphone membranes with bicarbonate-buffered dialysate.

We didn´t include in the current study HD patients for whom erectile stimulation drugs (phosphodiesterase inhibitors, alpha-receptors blockers, prostaglandins) or herbal were administered nor those under testosterone treatment. Were also not included patients receiving drugs that could induce hypothalamic-pitutary-gonadal axis dysregulations, those with acute or chronic hepatic disease as well as those with acute infections and clinical instabilities. On the basis of the given inclusion and non-inclusion criteria reported above, we selected a total of 55 male HD patients.

**Methods:** sociodemographic and clinical data (age, marital status, body mass index (BMI), predialysis blood pressure levels, etiology of ESRD, presence or not of diabetes, hypertension, and ischemic cardiopathy, seniority on dialysis, and erythropoietin treatment) were collected. Erectile function was assessed using the International Index of Erectile Function questionnaire (IIEF).

This multi-dimensional self-reported questionnaire evaluating male sexual function [17,18] was validated in 32 languages. It contains 15 items divided into 5 domains of sexual function: erectile function (6 items), intercourse satisfaction (3 items), orgasmic function (2 items), sexual desire (2 items), and overall satisfaction (2 items). According to the obtained score, erectile dysfunction is considered to be severe (6-10), moderate (11-16), mild to moderate (17-21) or mild (22-25). A score between 26 and 30 excludes the diagnosis of ED.

Biological parameters including phosphorus, calcium, parathyroid hormone (PTH), hemoglobin, total and high-density lipoprotein (HDL) cholesterol, triglycerides, and nutritional and inflammatory markers (albumin and C-reactive protein (CRP)) were assessed using standard methods at the biochemistry department. Urea reduction ratio (URR) was used to evaluate the adequacy of HD treatment. Hypothalamic-hypopituitary-gonadal axis was explored by obtaining fasting early-morning plasma samples and assessing the levels of total testosterone (immunoluminescence), FSH, prolactin, and LH.

**Statistical methods:** statistical analysis was performed using the statistical software SPSS 21 (SPSS Inc, Chicago, IL, USA). Data were expressed as mean ± standard deviation, frequencies (number), and proportions (%). Means were compared by one-way analysis of variance (ANOVA). Comparisons between groups with testosterone levels were established by Student's T test. A p-value <0.05 was considered statistically significant. Binary logistic regression analysis was performed using testosterone deficiency as dependent variable and variables with a p-value <0.2 in the univariate analysis.

## Results

**Sociodemographic, clinical, and hormonal characteristics of study participants:** a total of 55 male patients undergoing HD for at least six months were included in the current study. The mean age of included patients was 65 ± 13.9 years (range 20-60). The causes of underlying renal disease in our patients were: 12 patients (21.8%) had diabetic nephropathy, 13 (23.6%) had hypertensive nephrosclerosis, five (9.2%) had chronic pyelonephritis, six (10.9%) had adult polycystic kidney disease, four (7.3%) had chronic glomerulonephritis, and 15 (27.3%) had unknown cause of ESRD. The mean seniority in dialysis was of 3.33 ± 3.65 years. Fifty-four (98%) patients had an arteriovenous fistula; only one had a central venous catheter. Twenty-seven (49%) patients were habitual smokers and 33 (60%) patients were on treatment with erythropoietin-stimulating agent (ESA).

**Figure 1:**
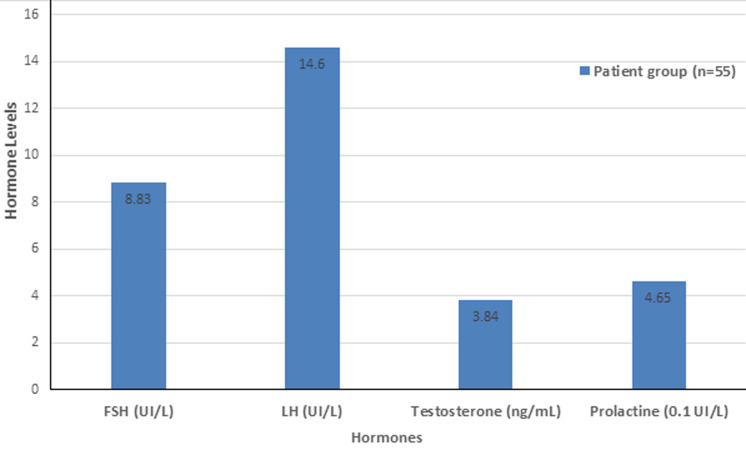
the distribution of testosterone, follicule-stimulating hormone (FSH), luteinizing hormone (LH) and prolactin level in hemodialysis (HD) patients

Hypotestosteronemia was noticed in 21 (38.2%) out of the 55 men undergoing HD. The mean level of testosterone in HD patients was of 3.84 ± ng/ml ± 1.77. The levels of FSH, LH, and prolactin were increased in patients undergoing HD. The distribution of testosterone, FSH, LH, and prolactin levels in the patients is shown in Figure 1.

**Figure 2:**
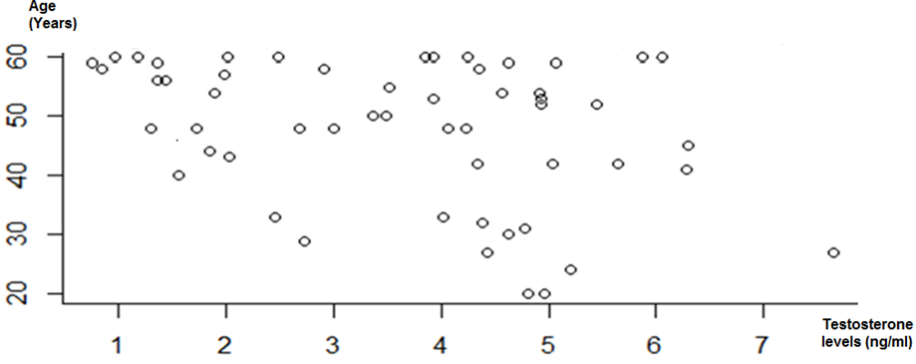
distribution of free testosterone with age

**Relationship between Testosterone levels, clinical and biological parameters:** testosterone was normal in younger patients and decreased in older ones. The oldest patients have the lowest testosterone levels (Figure 2).

Table 1 and Table 2 show the demographic, clinical, and biological parameters studied in all patients with testosterone levels above and below 3.4 ng/ml. Significant differences were observed in cases of advanced age, smoking, hypertension, obesity, seniority of dialysis, anemia, and absence of treatment by erythropoietin. The sample size did not allow for finding acceptable results in the multivariate analysis.

**Table 1:** demographic and clinical data at baseline by total testosterone

Background characteristics		Total population	Normal testosterone (≥3.46 ng/ml)	Low testosterone (<3.46 ng/ml)	p
Age (years)		48 ± 11.9	45.29	52.33	0.032
Smoking	Yes	28(50.9)	21	7	0.04
	No	27(40.1)	13	14	
Hypertension	Yes	27(49.1)	22	5	0.005
	No	28(50.9)	12	16	
Diabetes	Yes	45(81.8)	28	17	0.89
	No	10(18.2)	6	4	
Ischemic heart failure	Yes	36(65.5)	21	15	0.56
	No	19(34.5)	13	6	
BMI (kg/m^2^)		23.67± 4.31	22.57	25.44	0.015
SBP (mm Hg)		137.8± 21.1	140± 22.6	139± 20	0.38
Duration of renal failure (years)		6.02± 4.99	5.86	6.27	0.76
Duration of HD (years)		3.38± 3.67	2.86	4.22	0.18
Erythropoietin	Yes	33	15	18	0.004
	No	22	19	3	

BMI: body mass index; HD: Hemodialysis; SBP: systolic blood pressure

**Table 2:** laboratory distributions at baseline by total testosterone

Variables	Total population	Normal testosterone (≥3.46 ng/ml)	Low testosterone (<3.46 ng/ml)	p
URR (%)	70.4 ± 8.8	69.9±7.6	71.1±4.8	0.75
Kt/V	1.41 ± 0.38	1.42 ±0.41	1.43±0.37	0.58
Hemoglobin (g/dL)	9.32 ± 1.56	9.87	8.4	0.001
Atherogenic index (TC/HDL)	4.18 ± 2.01	4.26	4.05	0.71
PO4 (mmol/L)	1.85 ± 0.8	1.91	1.74	0.48
Calcium (mmol/L)	2.1 ± 0.28	2.06	2.17	0.17
PTH (pmol/L)	439.5 ± 268	407.5	491.09	0.3
C-reactive protein (mg/L)	14.14 ± 18	13.48	15.24	0.75
Albumin (g/L)	36.92 ± 7.3	37.53	35.84	0.45

Values for continuous variables are given as mean ± standard deviation. HDL: high density lipoprotein; Kt/v: K - dialyzer clearance of urea, t - dialysis time, V - volume of distribution of urea; PO4: serum phosphate; PTH: parathyroid hormone; TC: total cholesterol; URR: urea reduction

**Figure 3:**
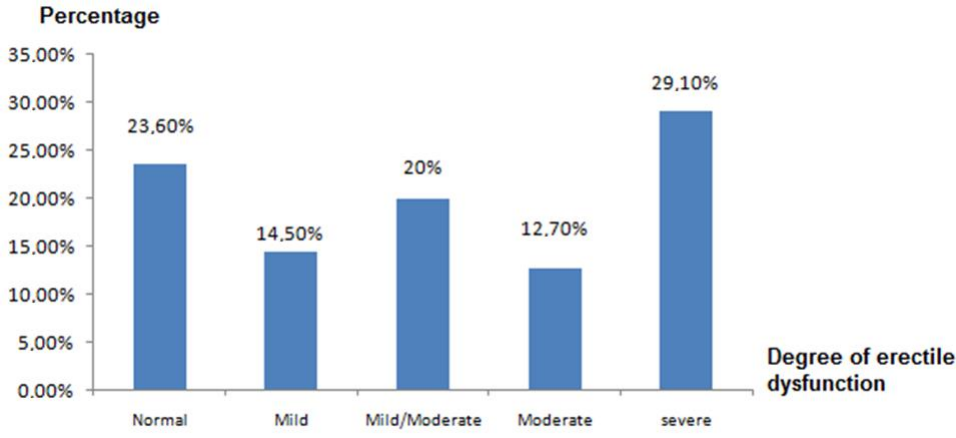
distribution of patients according to degree of erectile dysfunction (ED)

**Sexual disorder:** of the 55 men undergoing hemodialysis included in the study, 42 patients (76.4%) had erectile dysfunction. The mean of IIEF values was 12 ± 4. Figure 3 shows the different categories of erectile dysfunction in hemodialysis patients according to IIEF scores.

**Figure 4:**
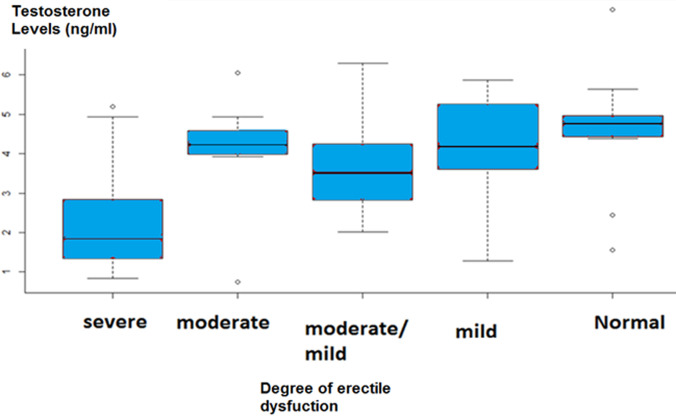
distribution of patients according to testosteronemia and erectile dysfunction

**Relationship between testosterone levels and erectile dysfunction:** serum testosterone was significantly decreased in patients with erectile dysfunction (Figure 4).

## Discussion

We have demonstrated through the current study that Hypogonadism was noticed in more than a third of patients. The levels of FSH, LH, and prolactin were increased in patients undergoing HD. These data are in accordance with those of literature showing that only 33.4% of patients with kidney failure requiring HD have a regular gonadal status [19-23].

Indeed, low testosterone level is a commonly described feature in ESRD patients. It has been established that over half of male renal failure patients have low or low-normal levels of testosterone [23-25] contrasting with 6-9% of affected men in the general population. HD patients have an accumulation of uremic toxins in many organs among which testis. This could negatively impact the secretion of testosterone by Leydig cells and induce hypotestosteronemia which in turn may cause an increase of gonadotropins via a positive retrocontrol on the hypothalamic-pituitary axis. Indeed, low testosterone levels may trigger pituitary cells to stimulate the secretion of FSH and LH. The increase in FSH levels could also be attributed to the inhibitory effect of inhibin secretion on the pituitary gland. According to literature data focusing on prolactin levels in HD patients, the increase of that hormone is a common feature in HD patients. As kidneys play a limited role in prolactin catabolism, the observed increase in prolactin levels could be explained by an excess of secretion by pituitary cells [26].

We have shown that factors classically related to hypogonadism in the general population, such as advanced age, obesity, hypertension [27,28], and smoking [29] had the same significance in patients undergoing hemodialysis. Indeed, we pointed out through the current findings a relationship between increasing age and serum testosterone decrease and this has been revealed in non-renal patients [30,31]. Many factors could explain the low testosterone level in aged patients. First, the production of GnRH diminishes in the elderly. Second, androgen-negative feedback suppression of LH secretion may be increased. Third, the diurnal rhythm of testosterone levels is not maintained with age [32]. Hypertension and anemia are aggravated with hypogonadism. Studies suggest that replenishment of testosterone to normal levels in hypogonadal men results in decreased blood pressure [33]. Testosterone enhances the proliferation of erythroid burst-forming units and colony-forming units by stimulating specific nuclear receptors [34]. Adiposity and obesity perpetuate the metabolic syndrome which results in a further decrease in testosterone levels [33]. Whereas, smoking increases testosterone levels and smoking cessation increases in hypogonadism [35].

The association between low testosterone, anemia, and lower response to erythropoetin in HD patients was established in our study as well as other reports [36,37]. Carrero *et al*. evaluated the relationship between testosterone and anemia in HD male patients and found that low levels of testosterone are effectively related to anemia and reduced response to erythropoetin [37]. However, controversial results were shown by Ekart *et al*. [38].

The efficiency of testosterone therapy in dealing with anemia in dialysis patients has been already studied. In a prospective trial, Gaughan *et al*. randomly divided anemic dialysis patients into two groups: the first group received three times weekly erythropoietin whereas the second group was given equal doses of erythropoietin in addition to nandrolone decanoate weekly. Hemoglobin levels were improved in both groups but were significantly better in the second group [39,40]. The impact of testosterone supplementation in HD patients was also investigated by Teruel *et al*. who noticed an increase in both erythropoietin and hemoglobin levels [41].

Otherwise, hypotestosteronemia could have multiple clinical implications including sexual function. Erectile dysfunction (ED) is a common complication of chronic kidney disease (CKD). The present study proved the high prevalence of ED among men with ESRD undergoing dialysis, which is in accordance with many studies [42-47]. Other studies reported higher rates rising to 90% [42,48,49]. The origin of ED in HD is multifactorial and complex. The disturbance in the hypophyso-gonadal axis such as changes in levels of gonadotropins, testosterone, and prolactin remains the major reason for the genesis of these disorders [50]. Testosterone therapy was shown to be efficient in managing these disorders which highlights the significance of hypogonadism in their pathogenesis [7,51].

Cangüven *et al*. evaluated the effect of testosterone gel therapy on men on dialysis with ED and hypogonadism. The treatment significantly increased testosterone levels, decreased levels of gonadotropins, and significantly improved international index of erectile function (IIEF) scores and erectile function [52]. Chatterjee *et al*. also studied patients with hypogonadism and ED and reported that injections of testosterone improved the IEFF score [53]. Consequently, one of the perspectives of the current study is to evaluate the potential benefits of androgen therapy on HD Tunisian patients.

Although our study is one of the first Tunisian studies on infertility screening in HD patients, it has some limitations mainly related to its monocentric character and the relatively small sample size. Moreover, ED evaluation was performed using the IIEF score with no further examination or tests. Hypogonadism diagnosis was based on a single testosterone dosage. Meanwhile, a single dosage was shown to be reliable in estimating the annual testosterone level [54].

## Conclusion

According to our findings, almost half of male Tunisian patients with ESRD had hypogonadism. Advanced age, anemia, and absence of adjuvant by erythropoietin may constitute risk factors for the low testosterone level in HD patients. So, the effects of testosterone replacement treatment in ED merit further investigation. Clinicians should be more attentive to precociously identify hypogonadism in HD patients in order to improve their quality of life.

### What is known about this topic

- *The prevalence of erectile dysfunction in male hemodialysis patients;*
- *The risk factors of erectile dysfunction in male hemodialysis patients*.

### What this study adds

- *Relationship with erectile dysfunction and hypogonadism in male hemodialysis patients*.

## References

[1] Chopp RT, Mendez R (1978). Sexual function and hormonal abnormalities in uremic men on chronic dialysis and after renal transplantation. Fertil Steril.

[2] Palmer BF (2003). Sexual dysfunction in men and women with chronic kidney disease and end-stage kidney disease. Adv Ren Replace Ther.

[3] Handelsman DJ, Dong Q (1993). Hypothalamo-pituitary gonadal axis in chronic renal failure. Endocrinol Metab Clin North Am.

[4] Gómez F, de la Cueva R, Wauters JP, Lemarchand-Béraud T (1980). Endocrine abnormalities in patients undergoing long-term hemodialysis. The role of prolactin. Am J Med.

[5] van Eps C, Hawley C, Jeffries J, Johnson DW, Campbell S, Isbel N (2012). Changes in serum prolactin, sex hormones and thyroid function with alternate nightly nocturnal home haemodialysis. Nephrology (Carlton).

[6] Prem AR, Punekar SV, Kalpana M, Kelkar AR, Acharya VN (1996). Male reproductive function in uraemia: efficacy of haemodialysis and renal transplantation. Br J Urol.

[7] Fiuk JV, Tadros NN (2019). Erectile dysfunction in renal failure and transplant patients. Transl Androl Urol.

[8] De Vries CP, Gooren LJ, Oe PL (1984). Haemodialysis and testicular function. Int J Androl.

[9] Levitan D, Moser SA, Goldstein DA, Kletzky OA, Lobo RA, Massry SG (1984). Disturbances in the hypothalamic-pituitary-gonadal axis in male patients with acute renal failure. Am J Nephrol.

[10] Schmidt A, Luger A, Horl WH (2002). Sexual hormone abnormalities in male patients with renal failure. Nephrol Dial Transplant.

[11] Dunkel L, Raivio T, Laine J, Holmberg C (1997). Circulating luteinizing hormone receptor inhibitor(s) in boys with chronic renal failure. Kidney Int.

[12] Iglesias P, Carrero JJ, Díez JJ (2012). Gonadal dysfunction in men with chronic kidney disease: clinical features, prognostic implications and therapeutic options. J Nephrol.

[13] Impotence (1992). NIH Consens Statement.

[14] Massaccesi L, Melzi d'Eril GV, Colpi GM, Tettamanti G, Goi G, Barassi A (2014). Levels of human erythrocyte membrane-bound and cytosolic glycohydrolases are associated with oxidative stress in erectile dysfunction patients. Dis Markers.

[15] (2000). iPLesp BiostaTGV.

[16] Skiba R, Matyjek A, Syrlyo T, Niemczyk S, Rymarz A (2020). Advanced Chronic Kidney Disease is a Strong Predictor of Hypogonadism and is Associated with Decreased Lean Tissue Mass. Int J Nephrol Renovasc Dis.

[17] Rosen RC, Riley A, Wagner G, Osterloh IH, Kirkpatrick J, Mishra A (1997). The international index of erectile function (IIEF): a multidimensional scale for assessment of erectile dysfunction. Urology.

[18] Barassi A, Pezzilli R, Morselli-Labate AM, Dozio E, Massaccesi L, Ghilardi F (2015). Evaluation of high sensitive troponin in erectile dysfunction. Dis Markers.

[19] Handelsman D (1985). Hypothalamic-pituitary gonadal dysfunction in renal failure, dialysis and renal transplantation. Endocr Rev.

[20] Singh AB, Norris K, Modi N, Sinha-Hikim I, Shen R, Davidson T (2001). Pharamacokinetics of a transdermal testosterone system in men with end-stage renal disease receiving maintenance hemodialysis and healthy hypogonadal men. J Clin Endocrinol Metab.

[21] Lim VS, Fang VS (1975). Gonadal dysfunction in uremic men. A study of the hypothalamo-pituitary-testicular axis before and after renal transplantation. Am J Med.

[22] Elbardisi H, Majzoub A, Daniel C, Al Ali F, Elesnawi M, Khalafalla K (2021). Endocrine contribution to the sexual dysfunction in patients with advanced chronic kidney disease and the role of hyperprolactinemia. Andrologia.

[23] Lundy SD, Vij SC (2019). Male infertility in renal failure and transplantation. Transl Androl Urol.

[24] Gungor O, Kircelli F, Carrero JJ, Asci G, Toz H, Tatar E (2010). Endogenous testosterone and mortality in male hemodialysis patients: is it the result of aging?. Clin J Am Soc Nephrol.

[25] Albaaj F, Sivalingham M, Haynes P, McKinnon G, Foley RN, Waldek S (2006). Prevalence of hypogonadism in male patients with renal failure. Postgrad Med J.

[26] Maciel GC, de Oliveira Antunes L, Chaves VA, Coelho OC, Nunes VG, Capuzi ER (2023). Hypogonadism and erectile dysfunction in patients with chronic kidney disease undergoing hemodialysis. World Journal of Advanced Research and Reviews.

[27] Bain J (2010). Testosterone and the aging male: to treat or not to treat?. Maturitas.

[28] Garibotto G, Esposito P, Picciotto D, Verzola D (2021). Testosterone Disorders and Male Hypogonadism in Kidney Disease. Semin Nephrol.

[29] Jones TH (2010). Testosterone deficiency: a risk factor for cardiovascular disease?. Trends Endocrinol Metab.

[30] Synder PJ (2001). Effects of age on testicular function and consequences of testosterone treatment. J Clin Endocrinol Metab.

[31] Purifoy FE, Koopmans LH, Mayes DM (1981). Age differences in serum androgen levels in normal adult males. Hum Biol.

[32] Khera M, Broderick GA, Carson CC, Dobs AS, Faraday MM, Goldstein I (2016). Adult-Onset Hypogonadism. Mayo Clin Proc.

[33] Fahed AC, Gholmieh JM, Azar ST (2012). Connecting the Lines between Hypogonadism and Atherosclerosis. Int J Endocrinol.

[34] Ferrucci L, Maggio M, Bandinelli S, Basaria S, Lauretani F, Ble A (2006). Low testosterone levels and the risk of anemia in older men and women. Arch Intern Med.

[35] Laaksonen DE, Niskanen L, Punnonen K, Nyyssönen K, Tuomainen TP, Valkonen VP (2005). The metabolic syndrome and smoking in relation to hypogonadism in middle-aged men: a prospective cohort study. J Clin Endocrinol Metab.

[36] Carrero JJ, Stenvinkel P (2012). The vulnerable man: impact of testosterone deficiency on the uraemic phenotype. Nephrol Dial Transplant.

[37] Carrero JJ, Bárány P, Yilmaz MI, Qureshi AR, Sonmez A, Heimbürger O (2012). Testosterone deficiency is a cause of anaemia and reduced responsiveness to erythropoiesis-stimulating agents in men with chronic kidney disease. Nephrol Dial Transplant.

[38] Ekart R, Taskovska M, Hojs N, Bevc S, Hojs R (2014). Testosterone and hemoglobin in hemodialysis male and female patients. Artif Organs.

[39] Gaughan WJ, Liss KA, Dunn SR, Mangold AM, Buhsmer JP, Michael B (1997). A 6-month study of low-dose recombinant human erythropoietin alone and in combination with androgens for the treatment of anemia in chronic hemodialysis patients. Am J Kidney Dis.

[40] Skiba R, Rymarz A, Matyjek A, Dymus J, Wozniak-Kosek A, Syrylo T (2022). Testosterone Replacement Therapy in Chronic Kidney Disease Patients. Nutrients.

[41] Teruel JL, Marcén R, Navarro JF, Villafruela JJ, Fernández Lucas M, Liaño F (1995). Evolution of serum erythropoietin after androgen administration to hemodialysis patients: a prospective study. Nephron.

[42] Ali ME, Abdel-Hafez HZ, Mahran AM, Mohamed HZ, Mohamed ER, El-Shazly AM (2005). Erectile dysfunction in chronic renal failure patients undergoing hemodialysis in Egypt. Int J Impot Res.

[43] Arslan D, Aslan G, Sifil A, Cavdar C, Celebi I, Gamsari T (2002). Sexual dysfunction in male patients on hemodialysis: assessment with the International Index of Erectile Function (IIEF). Int J Impot Res.

[44] Kleinclauss F, Kleinclauss C, Bittard H (2005). Erectile dysfunction in renal failure patients and renal transplant recipients. Prog Urol.

[45] Rosas SE, Joffe M, Franklin E, Strom BL, Kotzker W, Brensinger C (2001). Prevalence and determinants of erectile dysfunction in hemodialysis patients. Kidney Int.

[46] Mekki MO, El Hassan KA, El Mahdi EM, Haroun HH, Mohammed MA, Khamis KH (2013). Prevalence and associated risk factors of male erectile dysfunction among patients on hemodialysis and kidney transplant recipients: a cross-sectional survey from Sudan. Saudi J Kidney Dis Transpl.

[47] Seck SM, Dahaba M, Diouf B, Cisse MM, Gueye S, Ka EF (2011). The burden of erectile dysfunction in dialysis patients in Senegal. Hemodial Int.

[48] Gorsane I, Amri N, Younsi F, Helal I, Kheder A (2016). Erectile dysfunction in hemodialysis patients. Saudi J Kidney Dis Transpl.

[49] Malekmakan L, Shakeri S, Haghpanah S, Pakfetrat M, Sarvestani AS, Malekmakan A (2011). Epidemiology of erectile dysfunction in hemodialysis patients using IIEF questionnaire. Saudi J Kidney Dis Transpl.

[50] Bao J, Yu Q, Yu H, Hao J, Liu J, Yao J (2011). Erectile dysfunction in male hemodialysis patients in China--one center experience. Clin Nephrol.

[51] Palmer BF (1999). Sexual dysfunction in uraemia. J Am Soc Nephrol.

[52] Cangüven O, Aykose G, Albayrak S, Goktas C, Horuz R, Yencilek F (2010). Efficacy of testosterone gel in the treatment of erectile dysfunction in hypogonadal hemodialysis patients: a pilot study. Int J Impot Res.

[53] Chatterjee R, Wood S, McGarrigle HH, Lees WR, Ralph DJ, Neild GH (2004). A novel therapy with testosterone and sildenafil for erectile dysfunction in patients on renal dialysis or after renal transplantation. J Fam Plann Reprod Health Care.

[54] Carrero JJ, Qureshi AR, Parini P, Arver S, Lindholm B, Bárány P (2009). Low serum testosterone increase mortality risk among male dialysis patients. J Am Soc Nephrol.

